# Autism as early neurodevelopmental disorder: evidence for an sAPPα-mediated anabolic pathway

**DOI:** 10.3389/fncel.2013.00094

**Published:** 2013-06-21

**Authors:** Debomoy K. Lahiri, Deborah K. Sokol, Craig Erickson, Balmiki Ray, Chang Y. Ho, Bryan Maloney

**Affiliations:** ^1^Department of Psychiatry, Indiana University School of MedicineIndianapolis, IN USA; ^2^Laboratory of Medical and Molecular Genetics, Indiana University School of MedicineIndianapolis, IN, USA; ^3^Institute of Psychiatric Research, Indiana University School of MedicineIndianapolis, IN, USA; ^4^Department of Neurology, Indiana University School of MedicineIndianapolis, IN, USA; ^5^Cincinnati Children’s Hospital Medical CenterCincinnati, OH, USA; ^6^Department of Radiology and Imaging Sciences, Indiana University School of MedicineIndianapolis, IN, USA

**Keywords:** Alzheimer’s-autism continuum, anabolic hypothesis, neurite overgrowth, cranial volume

## Abstract

Autism is a neurodevelopmental disorder marked by social skills and communication deficits and interfering repetitive behavior. Intellectual disability often accompanies autism. In addition to behavioral deficits, autism is characterized by neuropathology and brain overgrowth. Increased intracranial volume often accompanies this brain growth. We have found that the Alzheimer’s disease (AD) associated amyloid-β precursor protein (APP), especially its neuroprotective processing product, secreted APP α, is elevated in persons with autism. This has led to the “anabolic hypothesis” of autism etiology, in which neuronal overgrowth in the brain results in interneuronal misconnections that may underlie multiple autism symptoms. We review the contribution of research in brain volume and of APP to the anabolic hypothesis, and relate APP to other proteins and pathways that have already been directly associated with autism, such as fragile X mental retardation protein, Ras small GTPase/extracellular signal-regulated kinase, and phosphoinositide 3 kinase/protein kinase B/mammalian target of rapamycin. We also present additional evidence of magnetic resonance imaging intracranial measurements in favor of the anabolic hypothesis. Finally, since it appears that APP’s involvement in autism is part of a multi-partner network, we extend this concept into the inherently interactive realm of epigenetics. We speculate that the underlying molecular abnormalities that influence APP’s contribution to autism are epigenetic markers overlaid onto potentially vulnerable gene sequences due to environmental influence.

## BACKGROUND

Autism is a specific form of what is now termed autism spectrum disorder (ASD). ASD is characterized by deficits in communication and social interaction and by stereotypic and rigid behaviors. In addition, macrocephaly, cognitive impairment, and seizures can be associated with ASD. Other brain anatomical abnormalities in autism have been reported in the literature, along with differences in intracranial volumes (Aylward et al., 1999, 2002; McCaffery and Deutsch, 2005). Pathologically, macrocephaly due to brain enlargement in autism is likely due to cell adhesion dysfunction. The amyloid-β precursor protein (APP), which is better known in association with Alzheimer’s disease (AD), is a known cell adhesion and neurite pruning protein (Thinakaran and Koo, 2008; Nikolaev et al., 2009). The amyloidogenic pathway favors loss of function APP with sequential cleavage of APP by β-secretase (BACE1) resulting in neurotoxic amyloid-β (Aβ) peptides 40 and 42, the major components of cerebral amyloid plaques associated with brain atrophy found in AD. *Alternative cleavage* via the α secretase non-amyloidogenic pathway releases the non-amyloidogenic secreted APP α (sAPPα; Hardy, 2009), believed to have neurotrophic properties (Mattson, 1994; Ray et al., 2011). The expected plasma neuronal marker profile in AD is low sAPPα and high Aβ 40/42. The non-amyloidogenic pathway (represented by high sAPPα and low Aβ 40/42) may represent a gain of function toxicity associated with neurodevelopmental conditions including autism. Of particular interest to autism research, we (Sokol et al., 2006; Ray et al., 2011), and others (Bailey et al., 2008), have determined that the cleavage product of APP, sAPPα, is elevated in plasma from autistic subjects vs. neurotypical and mildly autistic subjects (Ray et al., 2011).

**FIGURE 1:**
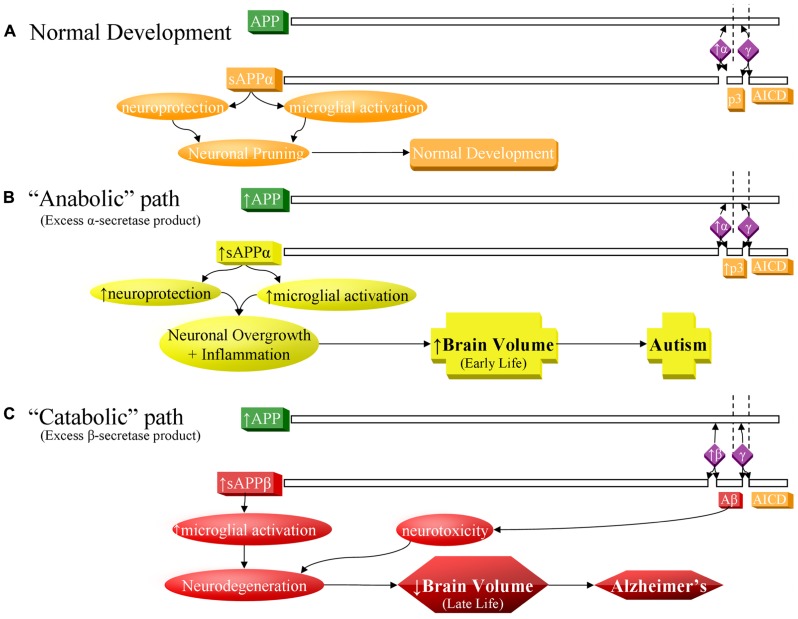
**Contrast of normal and pathogenic APP protein processing pathways.** **(A)** Normal pathway. APP protein (green) is processed by secretases (violet) and usually cleaved at the α-secretase site by ADAM10 or ADAM17 (α), then afterwards by the γ-secretase complex (γ). This produces sAPPα, the non-pathogenic p3 peptide product, and the APP intracellular domain (AICD). The sAPPα product is both neuroprotective and activates microglia. These balanced processes, under normal conditions, lead to neural pruning and normal development at appropriate times. This is the majority APP processing pathway. **(B)** APP anabolic dysfunction of the α-secretase pathway, specifically anabolic proteins/peptide levels, processes, and outcomes indicated in yellow. Overproduction of APP and/or excess α-secretase activity results in overproduction of sAPPα, which both activates neuroglia and is neuroprotective. Neuroprotective activity would presumably overwhelm microglial activation, since other molecules, presumably not also over-produced in this scenario, also activate microglia during neural pruning and would not be able to make up the difference to overwhelm additional sAPPα neuroprotection. The net result would bring about neuronal overgrowth and risk for autism. **(C)** The “catabolic”/neurodegenerative amyloidogenic β-secretase pathway, specifically catabolic protein/peptide levels, processes, and outcomes indicated in red. This pathway is associated with neurodegeneration and Alzheimer’s disease. Possible increase in APP protein and BACE1 protein levels (β) result in greater cleavage at the β-secretase and then by γ-secretase complex (γ). This produces sAPPβ, pathogenic/neurotoxic Aβ peptide, and AICD. sAPPβ activates microglia without offering neuroprotection. Thus, two of the three major products of the β-secretase pathway are neurodegenerative. If the β-secretase pathway becomes excessive, risk for Alzheimer’s disease increases.

These elevated sAPPα levels and increased intracranial volumes have led to the “anabolic hypothesis” of autism etiology:autism as a product of overgrowth (or insufficient pruning) of cranial neurons, resulting in neurological and behavioral symptoms (**Figure 1**). In addition to circumstantial evidence, specific APP pathways support this hypothesis. Fragile X syndrome (FXS), which is commonly marked by a comorbid diagnosis of ASD, involves the disruption of normal interaction between the fragile X mental retardation protein (FMRP) and metabotropic glutamate receptor (mGluR) which misregulates APP mRNA translation (Westmark and Malter, 2007). Of note, FXS has been associated with macrocephaly (Laxova, 1994). APP and metabolites also have a purported anabolic role within other translation regulating pathways such as Ras small GTPase/extracellular signal-regulated kinase (Ras/ERK; Venezia et al., 2006; Rohe et al., 2008) and phosphoinositide 3 kinase/mammalian target of rapamycin (P13K/mTOR; Rohe et al., 2008; Bhaskar et al., 2009).

The anabolic hypothesis of autism etiology provides specific molecular mechanisms for the development of ASD. Since the hypothesis is based upon levels of gene expression/translation, it has greater explanatory power than genetic mutation models. Likewise, recent years have seen greater recognition of environmental influences on gene expression, such as the enviromic/epigenomic latent early-life associated regulation (LEARn) model (Lahiri et al., 2009). Genetic predisposition would be conditioned by environmental risk to produce overall risk for autism and likewise point to potential preventative and treatment methods based on solid biochemical and neurological understanding of this disorder.

## AUTISM AS AN EARLY NEURODEVELOPMENTAL DISORDER

Autism is characterized by deficits in communication and social interaction and by stereotypic and rigid repetitive behaviors. Prevalence of ASD in the United States is estimated to be between 1 in 50 (Blumberg et al., 2013) and 1 in 88 (Autism and Developmental Disabilities Monitoring Network, 2012). Diagnosis can be made reliably as early as ages 18–24 months, with symptoms of autism seen as early as 9–12 months (Johnson and Myers, 2007). Infants with autism often show delayed onset of babbling, decreased gestures, imitation, and responsiveness within the first year. In the second and third years, there often is decreased frequency and diversity of language, less “showing” and “pretend” behaviors and reduced “joint attention,” considered a unique, core feature of autism (Landa, 2007). One in four children with autism may demonstrate word loss and other signs of communication regression during toddlerhood (Lord et al., 2004). Word loss stands out as a “red flag” and is considered unique to autism (Lord et al., 2004). This early presentation of symptoms suggests an associated prenatal/early childhood disruption of brain function that may underlie symptoms.

## APP IN EARLY BRAIN DEVELOPMENT

APP is a large (695–770 amino acid) glycoprotein produced in brain microglia, astrocytes, oligodendrocytes, and neurons (Mullan and Crawford, 1993). It has a large extracytoplasmic domain, a membrane-spanning domain containing the Aβ-peptide, and a short intracytoplasmic domain (Jolly-Tornetta et al., 1998). Mature APP is axonally transported and can be secreted from axon terminals in response to synaptic activation (Mattson, 1994) where it may play a role in neuronal maturation and synaptogenesis (Priller et al., 2006).

Proliferation, migration, differentiation, myelination, and synaptogenesis are all steps involved in generation of a mature neuron. Some of the known functions of APP in these processes include promotion of proliferation, cell–cell adhesion (Schubert et al., 1989), migration (Mattson, 1994), and synaptogenesis (Priller et al., 2006). More to the point, sAPPα has specific activity in inducing cellular proliferation (Siemes et al., 2004), including neural progenitor cells (Demars et al., 2011). sAPPα facilitates substrate adhesion in cell culture (Wehner et al., 2004). Induction of neuroprogenitor migration by sAPPα may be due to sAPPα upregulation of C–C chemokine levels (Vrotsos and Sugaya, 2009). sAPPα induces synaptogenesis in response to increases in ADAM10 (a disintegrin and metalloproteinase domain-containing protein 10; α-secretase) levels (Bell et al., 2008).

APP is predominantly located at synapses (Priller et al., 2006) and is released from neurons in an activity-driven fashion (Mattson and Furukawa, 1998). mGluR type 1 and type 5 (mGluR1/5) activation increases secretion of APP in cell culture (Jolly-Tornetta et al., 1998). The expression of APP appears to be developmentally controlled, with highest levels occurring early in synaptogenesis (Priller et al., 2006). APP levels are higher post-natally rather than prenatally but peak before 1 month of age in rodents (Lahiri et al., 2002). APP plays a functional role during growth cone development and has been implicated in neurite outgrowth (Mullan and Crawford, 1993; Mattson and Furukawa, 1998). APP works in opposition to NMDA (*N*-methyl-D-aspartate) and AMPA (α-amino-3-hydroxy-5-methyl-4-isoxazolepropionic acid) receptors with respect to glutamate’s pruning effects on growth cones (Mattson and Furukawa, 1998). Notably, APP blocks and reverses the ability of glutamate to inhibit dendrite outgrowth in embryonic rat hippocampal cell cultures (Mattson, 1994).

Knockdown of APP inhibits neuronal migration from the cortical ventricular zone to the cortical plate in mice (Young-Pearse et al., 2007). Conversely, overexpression of APP accelerates migration of neuronal precursor cells into the cortex (Young-Pearse et al., 2007). In cell culture, APP has been linked to suppression of neuronal cell adhesion (Schubert et al., 1989). Therefore, the location of APP at the synapse and its developmental function in migration and suppression of cell adhesion support the hypothesis that dysregulated levels of APP contribute to unguided brain growth as seen in autism (Courchesne et al., 2003). APP’s location at synaptic dendrites, its regulation by translation repressors such as FMRP, its participation in post-translational modification, and its role in neurogenesis and migration make it a prime candidate to contribute to the synaptic disruption in autism.

## PROCESSING OF APP

Amyloid-β precursor protein is best known in association with AD. Sequential cleavage of APP by the β-secretase, β-site APP cleaving enzyme 1 (BACE1) and the γ-secretase complex (presenilin 1, presenilin 2, nicastrin, anterior pharynx defective 1 homolog A, and other uncharacterized subunits) releases sAPPβ, the APP intracellular domain (AICD) and amyloidogenic Aβ peptide (**Figure 1C**), the major component of extracellular plaques found in AD (Thinakaran and Koo, 2008).

Although APP-related research is generally in the context of AD pathogenesis, the non-amyloidogenic (non-Aβ) “alpha” route is actually the predominant pathway for APP processing (Postina, 2008). Its rate-limiting enzyme is the α-secretase family of “a disintegrin and metalloprotease” (ADAM) proteins (ADAM9, 10, and 17; Deuss et al., 2008; Vingtdeux and Marambaud, 2012). In addition to potential “anti-pruning” activity, ADAM17 induces cellular proliferation in a variety of conditions (Gooz et al., 2009; Lin et al., 2012). Release of sAPPα is complete by the γ-secretase complex (Lahiri et al., 2003; Thinakaran and Koo, 2008), along with the non-amyloidogenic 3 kDa “p3” peptide and the AICD (**Figure 1A**). sAPPα exhibits a wide array of neurotrophic activities (Mattson, 1997; Turner et al., 2003), important for neurodevelopment. sAPPα activates microglia, a function it shares with sAPPβ (Barger and Harmon, 1997). In mice, sAPPα increases neurite outgrowth and memory and protects against multiple insults (Stein and Johnson, 2003). It has been suspected that APP contributes to the predisposition to hematologic malignancy in Down syndrome patients and that APP aberration might predispose to cancer (Morris et al., 2010). APP is among the most overexpressed genes in acute myeloid leukemia patients with complex karyotypes (Baldus et al., 2004) and in solid tumors (Arvidsson et al., 2008; Krause et al., 2008). Promotion of the non-amyloidogenic pathway may be a promising novel treatment in AD (Bandyopadhyay et al., 2007). Recently, there has been interest in the function of sAPPα in neurodevelopment and its relationship to autism (Sokol et al., 2006) and FXS (Westmark and Malter, 2007). No specific tie has been found between p3 or AICD and autism.

ADAM17 is also involved in the processing of tumor necrosis TNF-α (tumor necrosis factor-alpha) at both the cell surface and within the trans-Golgi network (Black et al., 1997). This process, known as ”shedding,” involves cleavage and release of a soluble ectodomain from membrane-bound pro-proteins (such as pro-TNF-α), and is of known physiological importance (Black et al., 1997), particularly in neoplastic proliferation (Xiao et al., 2012; Zheng et al., 2012). While ADAM17 itself has not been specifically studied in association with autism, the cerebrospinal fluid to serum ratio for TNF-α is elevated in subjects with autism compared to other pathological states (Chez et al., 2007). Furthermore, linkage was found between a region of human chromosome 2 that contains the ADAM17 gene and autism (Allen-Brady et al., 2010).

## GENETICS OF AUTISM

Twin studies, family studies, and predominately male distribution of the disorder attest to the heritability of autism. Twin studies have consistently shown that monozygotic twins are up to 10 times more likely to be concordant for autism than are dizygotic (Folstein and Rutter, 1977; Steffenburg et al., 1989; Bailey et al., 1995). This rate can be evaluated in comparison to other neurobiological disorders such as AD, depression, bipolar disorder, and schizophrenia (between 2:1 and 4:1), indicating a high heritability for autism (Gatz et al., 2006; Pennington, 2009). However, the significantly earlier-life pathogenesis of autism vs. these other disorders means that one cannot automatically exclude environmental “protective” influences potentially reducing net genetic influence on heritability in AD, schizophrenia, etc. Family studies (Piven and Palmer, 1999; Rutter, 2000) suggest that the risk of autism is 20–60% higher in siblings compared to in the general population. In addition, first degree relatives of individuals with autism were shown to be shy, aloof, and have problematic pragmatic language (Rutter, 2000), consistent with segregation of sub-threshold traits within these families (Abrahams and Geschwind, 2008). These and other associations have led to the definition of a “broad autism phenotype,” which would suggest classifying autism as extreme manifestation of a normal human variation (Sucksmith et al., 2011) rather than inherently pathological in all its traits.

Autism affects more boys than girls (4:1), a finding that has remained consistent since Kanner’s first description in 1945, despite the increasing prevalence of its diagnosis. The predominantly male ratio has been attributed to abnormality on the X chromosome, or to sex linkage or genomic imprinting (Marco and Skuse, 2006). However, such linkage has not been found in all cases of autism, suggesting the importance of genetic pathways other than the X chromosome. With rare exceptions, however, autism does not appear to be the action of a single gene inherited in a strictly Mendelian pattern, be it autosomal dominant, autosomal recessive, or X-linked (O’Roak and State, 2008).

So-called “rare” genetic variants may contribute to autism (Vorstman et al., 2006), although “rare” variants (disregarding specific disease associations) are overall quite common, with an estimated frequency of up to 3.4 such variants per person (Nelson et al., 2012). Cytogenetic, gene association, linkage, microarray technology, copy number variation (CNV) analysis and exome sequencing lead to estimates of chromosomal abnormalities in autism that range from 6 to40% (Marshall et al., 2008; Pennington, 2009; Abrahams and Geschwind, 2010). Chromosome regions associated with autism include chr2q37, chr7q22, chr10q23, chr17q11-21, chr22q11, chr22q13, and chr15q11-13 (Sokol and Lahiri, 2011). Non-additive interaction among genes (epistasis) has been proposed to account for such a large range of chromosomal abnormality estimation (Poot et al., 2011; Ruzzo et al., 2012). Of course, this leads to the inescapable question, what accounts for the remaining 94–60% of autism not associated with chromosomal abnormalities? While numerous studies identifying candidate genes or markers have been reported, very few have been replicated (Losh et al., 2008). One solution to this problem is to study endophenotypes associated with autism (Duvall et al., 2007; de Geus, 2010). None of the endophenotype studies has produced a “definitive” solution. Instead of continuing down a single-cause (DNA mutation) path, it may be more useful to presume that autism is a complex disorder that depends upon interaction of multiple levels of organization, not amenable to simple genetic modeling.

## BRAIN ABNORMALITY IN AUTISM: HISTOLOGY AND *POST-MORTEM* ANATOMY

Little to no abnormality is revealed by standard hematoxylin and eosin staining of autistic brain tissue (Casanova, 2007). Complex assessment is necessary to reliably determine differences, and research has required many samples to separate autistic from controls. *Post-mortem* brain studies of autism have been hindered by small sample sizes, with fewer than 150 autism cases studied to date, the majority of them adults. Therefore, the peak aberrant neurological growth identified by magnetic resonance imaging (MRI) studies has not yet been confirmed via neuropathology (Schumann and Nordahl, 2011). Often, findings cannot be repeated because the numbers of deaths is low in this group, and death is often due to a cause that is likely to have an effect on the histopathology, e. g., seizures.

These difficulties notwithstanding, in *post-mortem* studies, evidence of autism-associated brain pathology includes disordered interregional connectivity (Courchesne and Pierce, 2005), including reduction in the size of the corpus callosum (Egaas et al., 1995), minicolumn pathology (Casanova et al., 2006), deranged neuronal development (Bauman and Kemper, 1985; van Kooten et al., 2008), and brain cytoarchitecture (Bauman and Kemper, 1985), and irregularity in brain structures associated with social behavior (Stanfield et al., 2008; Wegiel et al., 2010). Macrocephaly is one of the most widely replicated biological findings in autism, affecting up to 20% of children with the condition (Aylward et al., 1999, 2002; McCaffery and Deutsch, 2005) and confirmed by MRI volumetric studies, described below (Piven et al., 1996; Courchesne et al., 2001; Aylward et al., 2002; Sokol and Edwards-Brown, 2004) and increased brain weight (Bauman and Kemper, 1985; Bailey et al., 1993). While occipital-frontal head circumference (OFC) appears normal at birth, excessive brain growth occurs early, around the time symptoms appear. Later growth may plateau or decline to normal circumference in adulthood (McCaffery and Deutsch, 2005). Proposed mechanisms underlying brain enlargement include overproduction of synapses, failure of synaptic pruning, excessive neurogenesis and gliogenesis, or reduction in cell death (McCaffery and Deutsch, 2005)

Pathological studies of the frontal lobes of autistic brains, which subserve social relatedness, the ability to change set, and the persistence of repetitive behaviors, show increased microglial activation (Vargas et al., 2005), and volume (Morgan et al., 2010), indicating potential neuroinflammation. Frontal lobe spindle neurons (Schumann and Nordahl, 2011), showed no differences in adults (Kennedy et al., 2007), but there was an increased ratio of spindle neurons to pyramidal neurons in children (Santos et al., 2011). In adults, a poorly defined boundary was found at the frontal, parietal, and temporal lobe gray-white matter junctions, suggestive of abnormalities in neurogenesis or neuronal migration (Avino and Hutsler, 2010).

Minicolumns, also known as microcolumns, are vertical columns of neurons organized into pathways and intrinsic circuits with a similar receptive field (Buxhoeveden et al., 2001, 2006). Casanova et al. (2002)found significant differences between the brains of autistic patients and controls in the number of minicolumns, in the horizontal spacing that separates cell columns, and in their internal structure. These findings have been essentially replicated (Buxhoeveden et al., 2006; Casanova et al., 2006) and indicate that excess proliferation of neurons early in development is followed by a decrease in dendritic arborization, accounting for early overgrowth followed by normal brain volume in autism (Schumann and Nordahl, 2011). However, Casanova et al.’s (2007) later comparison of the minicolumns of three internationally distinguished neuroscientists vs. six non-scientist controls showed a minicolumn pattern similar to what they found for autism, although none of the scientists had reported autism-like symptomology. This was explained by noting that narrower minicolumns may favor discrimination and focused attention, two traits necessary for scientific achievement. On the other hand, autistic brains also have poorly synchronized and weak connectivity between brain regions. For example weak connectivity has been reported between the frontal cortex that supports decision making, the left temporal lobe that supports speech, and right temporal lobe that supports visual-spatial recognition (Courchesne and Pierce, 2005). This may explain how individuals with autism may lack facial recognition and have trouble verbally expressing their ideas. Human APP and *Drosophila* equivalent APPL can induce post-developmental axonal arborization in the *Drosophila* CNS after brain damage (Leyssen et al., 2005). Further, APP has been associated with neurogenesis and neuronal migration (Mattson and Furukawa, 1998). Of particular note, knockdown of APP alters recruitment of interneurons in L5 and affects their laminar distribution (Lodato et al., 2011), which would likewise alter organization and integration of neurons into functional neural circuits. While sAPPα has not yet been studied in regard to microcolumnar organization, amyloid-β deposits have been shown to contribute to loss of microcolumnar organization (Buldyrev et al., 2000). APP may play a role in neuronal network connectivity; it’s specific role in minicolumn development remains to be determined.

There has been much work on neuropathology in the amygdala, known to be involved with the perception of fear, anxiety, and obsession-compulsion that would interfere with social relations. Kemper and Bauman (1993)found unusually small, densely packed neurons in amygdale from older children and adults with autism compared to controls. Schumann and Amaral (2006)found fewer neurons in the total amygdala but no increase in neuronal density or decrease in the size of neurons from old children and adults. It has been speculated that an excessive number of neurons would be initially generated during early development with their subsequent elimination during adulthood (Schumann and Nordahl, 2011). This could explain inconsistent findings between the neuropathology studies and the amygdala enlargement reported in the brain MRIs of younger children with autism (Sparks et al., 2002; Schumann et al., 2004; Schumann and Amaral, 2006).

## BRAIN ABNORMALITY IN AUTISM: FINDINGS FROM MAGNETIC RESONANCE IMAGING

Brain MRI studies indicate that very young children with autism (ages 18 months to 4 years) have a 5–10% abnormal enlargement in total brain volume (Courchesne et al., 2001, 2003; Sparks et al., 2002), although increased size of the adult brain also has been reported (Piven et al., 1996). In addition, cross-sectional and longitudinal specific enlargement of the frontal and temporal lobes has been found in 2 year olds with autism (Schumann et al., 2010), and increased size of the adult brain also has been reported (Piven et al., 1996). The amygdala also undergoes developmental enlargement in young boys with autism (Sparks et al., 2002; Schumann et al., 2004) and later follows a growth trajectory different from controls. A recent longitudinal study of 38 children with autism and 21 controls showed enlargement of cortical volume (surface area), but not cortical thickness at age two compared to ages 4 and 5 (Hazlett et al., 2011). Cortical surface area was linked to the number of minicolumns in the cortical layer (Rakic, 1988), while cortical thickness is thought to reflect dendritic arborization (Huttenlocher and Hapke, 1990).

## MECHANISMS OF APP IN AUTISM

We have previously reported high levels of total plasma sAPP (including sAPPα) in a small sample of young children with severe autism and aggression (Sokol et al., 2006). These children expressed sAPP at two or more times the levels of children without autism and up to four times more than children with mild autism. Overall, there was a trend towards higher levels of both sAPPα and total sAPP in children with autism, combined with a non-significant decrease in Aβ40. This pointed toward the possibility of increased non-amyloidogenic (growth-promoting or anabolic) processing in autism. These findings have been replicated and extended by an independent laboratory: Elevated plasma sAPPα was found in 60% of known autism children (*n* = 25) compared to healthy age-matched controls (Bailey et al., 2008). A recent follow up by our laboratory in a separate, larger set of autism and control patient plasma samples (16 autism, 18 control), confirmed the original finding of significantly elevated sAPPα in plasma of severe autism patients, although without coexistant aggression (Ray et al., 2011). Elevation in sAPPα was not found with mild autism in either study. Crucially, this work showed a decrease in levels of both Aβ40 and Aβ42 in severely autistic patients compared to controls.

In summary, APP is regulated by FMRP via the mGluR receptor (Westmark and Malter, 2007). Functional consequences of excessive mGluR signaling in absence of FMRP include prolongation of epileptic form bursts in hippocampal area CA3 (Bailey et al., 2008), elongation of dendritic spines on cultured hippocampal neurons (Vanderklish and Edelman, 2002) and long-term depression (LTD) in hippocampal area CA1 (Li et al., 2007). These findings are associated with the FXS clinical phenotypes: epilepsy, elongated and immature dendritic spines, and cognitive delay, according to the mGluR theory of FXS (Bear et al., 2004).

If mGluR5 signaling is enhanced in FXS individuals, excessive APP translation would be expected, inevitably leading to higher sAPPα levels. As evidence shows that excessive mGluR5 signals favors an excitatory, anabolic state in FXS, we speculate that overexpression of the mGlur5 pathway and resultant higher sAPPα levels may likewise contribute to aggression, seizures, and intellectual deficit seen in severe autism. Further, it is hypothesized that such anabolism may contribute to brain overgrowth associated with autism.

One mechanism by which sAPPα could contribute to brain overgrowth is by disrupting cell adhesion. Hazlett et al. (2011)showed that early brain enlargement typical of autism may be associated with increased surface area overgrowth due to faulty cell adhesion. One such mechanism would be faulty adhesion molecule β-catenin, a component of the cadherin protein complex that constitutes adherens junctions. Adhesion molecules are thought to reduce growth of brain progenitor cells. APP modulates β-catenin degradation *in vitro* and *in vivo* (Chen and Bodles, 2007). Evidence also suggests that molecular defects in autism interfere with synaptic protein synthesis (Kelleher and Bear, 2008). Defects in translational repression would favor an anabolic state, underlying the autistic phenotypes of macrocephaly, cognitive impairment, and seizures.

## APP AND FMRP

Protein synthesis is reduced in FXS, a rare neurodevelopmental condition (1 in 4000 males and 1 in 10,000 females; National Fragile X Foundation, 2013), associated with intellectual disability, and ASD in 2 in 3 males with FXS (Hatton et al., 2006). FXS is caused by a trinucleotide repeat (CGG repetitive sequence) in the promoter region of the fragile X mental retardation 1 gene (FMR1). This gene’s product, FMRP, is important for normal brain development. FMRP is an RNA binding and carrier protein that carries the messages produced from many other genes to the synapse. FMRP is involved in both activity-dependent transport of target mRNAs and in regulation of local protein synthesis at the synapse (Bagni and Greenough, 2005). Local protein synthesis following synaptic activity is necessary for maintenance of some plastic changes at the synapse and likely it is important for changes in spine morphology (Grossman et al., 2006). Therefore, FMRP-mediated regulation of local protein synthesis is presumably essential for normal memory and learning.

Fragile X mental retardation protein can be synthesized locally in proximal dendrites (Feng et al., 1997), or recruited to the synapse from more distant sites after mGluR activation (De Diego Otero et al., 2002). mGluR1/5 receptors are positioned in the post-synaptic membrane, where they activate a Gq-coupled second messenger system that transduces glutamate release into downstream phosphorylation cascades. Activation can lead to either long-term potentiation (LTP) or LTD depending on cell type and brain location. Activation of mGluR5 releases FMRP-mediated translation repression and results in protein synthesis-dependent LTD (Bear et al., 2004). Functional consequences of excessive mGluR signaling in absence of FMRP include prolongation of epileptic form bursts in hippocampal area CA3 (Bailey et al., 2008), elongation of dendritic spines on cultured hippocampal neurons (Vanderklish and Edelman, 2002), and LTD in hippocampal area CA1 (Li et al., 2007). These findings are associated with the FXS clinical phenotypes: epilepsy, elongated, and immature dendritic spines, and cognitive delay, according to the mGluR theory of FXS (Bear et al., 2004).

In the resting state, FMRP binds to and inhibits dendritic translation of up to 4% of brain mRNAs including APP (De Rubeis and Bagni, 2010). APP is regulated by FMRP via the mGluR receptor (Westmark and Malter, 2007). If mGluR5 signaling is enhanced in autistic individuals, excessive APP translation would be expected, inevitably leading to higher sAPPα levels. Recently, we found elevated sAPPα in the plasma of children with FXS (*n* = 18) compared to typically developing, age-matched controls (*n* = 18; Lahiri et al., 2011). Further, levels of Aβ40 and Aβ42 were higher in FXS compared to controls (Lahiri et al., 2011). Preliminary evidence for this same pattern, i.e., high levels of sAPPα and Aβ, was found for a very small sample of left temporal lobe brain tissue of FXS (*n* = 2) compared to typically developing, age-matched control (*n* = 1). These results suggest investigating whether FXS receives a “double dose” of deleterious components from non-amyloidogenic and amyloidogenic pathways. Aβ40 and Aβ42 levels were significantly higher in two strains of fmr-1 knockout mice compared to wild type (Westmark and Malter, 2007). Genetic downregulation of mGluR5 signaling has reversed behavioral deficits in fmr-1 knockout mice (Dolen et al., 2007; Dolen and Bear, 2008). Simple mGluR5 antagonism, predicted to reduce APP via the anabolic pathway (Sokol et al., 2011), may reverse these effects in humans.

## APP IN THE Ras/ERK AND PI3K/Atk/mTOR PATHWAYS

The Ras small GTPase/extracellular signal-regulated kinase (Ras/ERK) and phosphoinositide 3 kinase/protein kinase B/mammalian target of rapamycin (PI3K/Akt/mTOR) signaling pathways pair synaptic activity to the translational machinery and are also involved in protein synthesis-dependent LTP and LTD (Kelleher and Bear, 2008). Mutation of the proteins which regulate these pathways associates with high prevalence of autism and intellectual deficit (Levitt and Campbell, 2009). Inactivating mutations of several negative regulators of the ERK and mTOR pathways, such as neurofibromin (neurofibromatosis type 1), harmartin, and tuberin (tuberous sclerosis), and PTEN (phosphatase and tensin homolog) are responsible for genetic disorders with a high prevalence of cognitive impairment and autism (Levitt and Campbell, 2009). In the absence of functional proteins, these pathways are “turned on” to excess. These signaling pathways are activated not only by mGluR receptors, but also NMDA and neurotrophin Trk receptors (Kelleher and Bear, 2008). Ras/ERK is activated by sAPPα (Demars et al., 2011), its secretase ADAM17 (Diaz-Rodriguez et al., 2002), and neurotrophin Trk receptors. TrkB has the highest affinity to the binding of brain-derived neurotrophic factor (BDNF), a growth factor with important roles in the survival and function of neurons and linked to both ASD and AD (Nickl-Jockschat and Michel, 2011). Indeed, effects of acamprosate (*N*-acetyl-homotaurine) on behavior and BDNF recently has been studied in youth with FXS (Erickson et al., 2013). The researchers suggested that the increased BDNF levels with treatment may serve as a useful pharmacodynamic marker, which is consistent with the proposed anabolic model. It would be interesting to test whether a change in sAPPα levels could serve as another important pharmacodynamic marker in neurodevelopmental disorders.

The PI3K/Akt/mTOR pathway is an FMRP-dependent pathway (Narayanan et al., 2008). PTEN is a negative regulator of the PI3K pathway. PTEN mutation phenotypes include brain tumors, macrocephaly, and autism (Butler et al., 2005; Kerr et al., 2006). There is evidence that sAPPα, specifically, induces cellular proliferation through the PI3K/mTOR pathway (Cheng et al., 2002), and sAPPα activates Akt (Demars et al., 2011).

## APP AND PROTEIN KINASE C

Reduced activity of protein kinase C (PKC) associates with regressive autism (Ji et al., 2012). A linear relationship has been noted between reduction in PKC activity and restricted, repetitive, and stereotyped behaviors (Ji et al., 2012). Likewise, specific haplotypes in the protein kinase c-β (PRKB1) gene are associated with autism (Philippi et al., 2005). Protein phosphorylation by kinases including PKC drives APP processing toward the anabolic α-secretase pathway (Buxbaum et al., 1990; Caporaso et al., 1992). This apparently contradicts the anabolic hypothesis and would require further study.

## THE UNIFYING EPIGENETIC LEARn MODEL IN AUTISM

Amyloid-β precursor protein’s role in FMRP, ERK, and mTOR pathways is consistent with an overall, pro-growth, anti-apoptotic role for APP. In a situation of nerve growth factor withdrawal, Aβ production is upregulated, leading to neuronal apoptosis (Matrone et al., 2008). In this way, APP activates both trophic (through sAPPα) and apoptotic (through Aβ) pathways, and the predominance of one may determine pathology: autism vs. AD. The finding that the same gene can promote anabolism and catabolism is reminiscent of FMRP’s role in FXS and Fragile X-associated tremor/ataxia syndrome (FXTAS) found in subsets of older adults harboring FMR1 premutations (Hagerman et al., 2001). FXTAS is a condition of progressive tremor and ataxia in individuals who show no pre-morbid cognitive deficits, developing over the age of 50. Dementia occurs in a subset of those with FXTAS. It is believed that FXS is caused by FMRP loss of function, and FXTAS is caused by an FMR1 mRNA gain of function toxicity (Sokol et al., 2011). In the case of APP, loss of function would favor the amyloidogenic pathway leading to AD while gain of function toxicity would favor the non-amyloidogenic pathway leading to excessive ADAM17, sAPPα, and brain overgrowth associated with autism.

Autism is a complicated disorder for which many models combining genetic and external factors have been proposed (Newschaffer et al., 2007; Abrahams and Geschwind, 2008; Pennington, 2009). A key point of the anabolic hypothesis is that, unlike many genetic models, it is not a “change of function” model. Genetic models based on coding sequence variation have generally presupposed that a pathogenic variant results in loss or gain of function for a protein. The anabolic hypothesis, on the other hand, does not propose qualitative differences in the activities of the participating molecules. Instead, it is a quantitative model. Variation in activity *levels* spells the difference between health and disorder. Qualitative mutation in associated proteins could be informative, particularly loss of function mutations, as these would effectively mimic variations that result in atypically low levels of a protein of interest. Likewise, gain of function mutations could essentially mimic effects of atypically high levels of a protein, which permit a minority function to reach a critical threshold.

Our basis for preferring an epigenetic explanation is a response to dead ends and results from purely genetic models that fell very far short of original expectations. Laying out matters plainly, no genetic model has proved adequate to explain non-syndromic autism, the condition’s most common form. Ever-finer, ever-broader GWAS or other genetic studies have repeatedly failed to find the magic target. As a result, models have had to resort to ever-more Byzantine multi-gene invocations (Allen-Brady et al., 2009; Anney et al., 2010). We propose an epigenetic model specifically in the face of the failure of purely genetic presumptions, which may have come to the point of invoking a “hidden variable” argument analogous to those used by opponents of quantum physics.

Many of the proteins implicated in the anabolic hypothesis are subject to or take part in epigenetic regulation. *FMR1* has multiple phenotypes depending upon variable DNA methylation (de Vries et al., 1996). Regulation of critical APP protein processing enzymes was altered by changes in DNA methylation (Fuso et al., 2005). ERK/mitogen-activated protein kinases (MAPK) signaling activity drives epigenetic modification that underlies stress, learning, and memory processes (Trollope et al., 2012). It is now accepted that epigenetic states can change after birth, including by age-related drift (Martin, 2005) and specific changes associated with late-life neurological disorders, such as AD (Poulsen et al., 2007; Wang et al., 2008). Epigenetic changes can occur in response to environmental stressors, such as exposure to heavy metals (Wu et al., 2008) and famine (Hughes et al., 2009; Martin-Gronert and Ozanne, 2010). Of particular interest is that these exposures can have occurred in the previous generation, before conception, with effects passed along to offspring (Flory et al., 2011). 

In addition to the already-mentioned effect of DNA methylation status on *FMR1*-associated phenotypes (de Vries et al., 1996), abnormal DNA methylation was found in the 5′-CpG island for the UBE3A gene of autistic subjects (Jiang et al., 2004). The oxytocin receptor gene has aberrant DNA methylation in its CpG island, depending upon autism status (Gregory et al., 2009). These specific differences were reflected in a wider scale across prefrontal cortex neurons, which showed changes in chromatin structure at multiple gene loci associated with neuronal connectivity, social behaviors, and cognition, with altered levels of corresponding transcripts (Shulha et al., 2012). Even the X chromosome association explanation for the predominance of male autistic patients has been questioned on epigenetic grounds. Specifically, sex hormone activity mediates epigenetic modifications of DNA and histones, increasing or decreasing risk of various diseases, such as autism (Kaminsky et al., 2006).

Finally, to address the function of APP-in the anabolic hypothesis, while evidence exists for levels of anabolic forms of APP (e.g., sAPPα) to contribute to autism, as we have outlined herein, no specific genetic link has as of yet been reported in the literature. We suggest that lack of discovery may be due to lack of presence. A testable alternative hypothesis to explain APP’s role would be epigenetic pathways.

Many workers have proposed that autism is a result of complex interaction between genetic and environmental factors (Newschaffer et al., 2007; Costa e Silva, 2008; Dufault et al., 2012). A specific, testable expression of such concepts would be the LEARn model (Lahiri et al., 2009; Maloney et al., 2012) in which complex neurological disorders require multiple “hits” to clinically manifest. Earlier hits would be latent epigenetic markers until sufficient critical hits are accumulated by a necessary life span cutoff point, at which time a disorder would become apparent. Should insufficient hits be suffered, or should hits be successfully detected and remediated before the developmental threshold occurs, no disease would appear.

In terms of autism, hits of interest could be those of early post-natal development, particularly any that turned out to be associated with DNA oxidation or DNA hypomethylation. These two particular environmentally induced gene sequence lesions have already been shown to be amenable to dietary remediation by addition of *S*-adenosyl methionine, which resulted in reversal of induced hypomethylation (Rogers et al., 2004; Chan and Shea, 2006; Howard et al., 2011; Jousse et al., 2011). Likewise DNA methylation status can be altered by social interaction, such as differences in maternal care and rearing practices (Szyf, 2007; Champagne and Curley, 2009; McGowan et al., 2009). LEARn-informed research could produce rational relationships for therapy and brain biochemistry in autism and early autistic conditions.

## CONCLUSION

**FIGURE 2:**
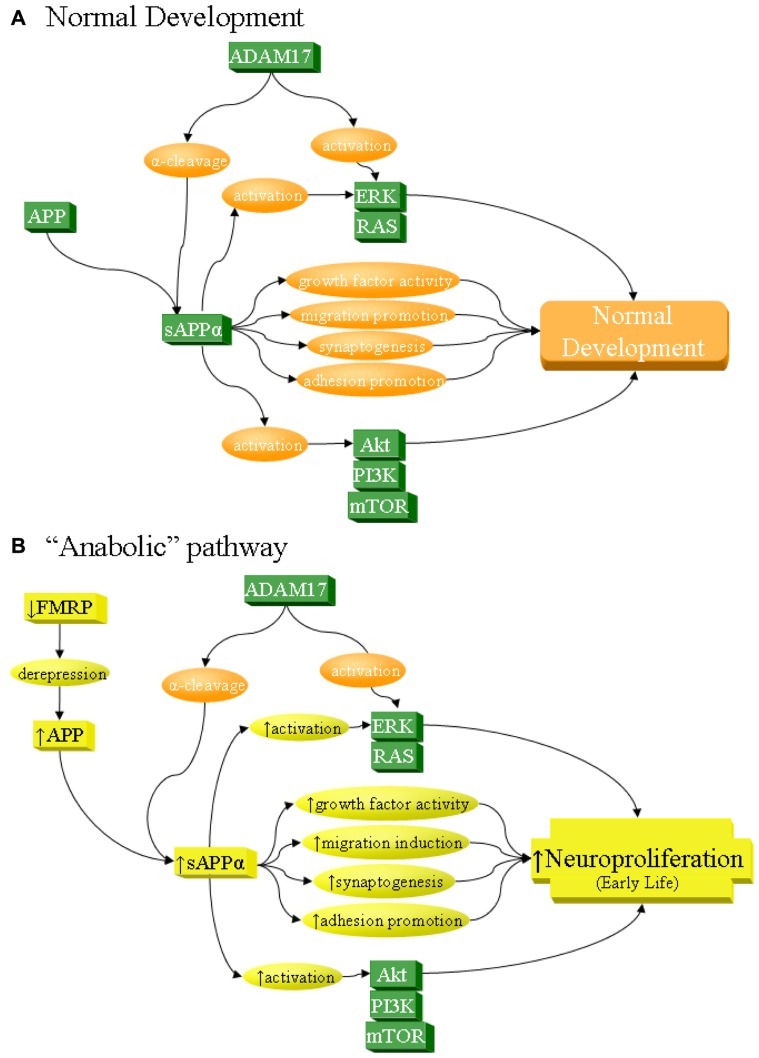
**sAPPα as a network activator in anabolic etiology of autism**. The sAPPα product has multiple functions that can contribute to neuronal overgrowth and increase risk of autism. Specifically, sAPPα, produced by α-secretase activity of proteins such as ADAM17, acts on its own as a cell growth factor, stimulates neuronal migration, induces synaptogenesis, and promotes cellular adhesion, all of which lead to neuroproliferation. In addition, sAPPα activates ERK and Akt, which in turn also lead to neuroproliferation. **(A)** Under normal development, these processes result in normal growth. **(B)** sAPPα can contribute to anabolic etiology of autism through several potential pathways, including overexpression of APP. Such overexpression could be from derepression via FMRP deficiency, as in FXS. It must be stressed that this is not the only possible cause of APP overexpression and is cited simply as one of many potential examples. Increase in APP would lead to greater sAPPα through the already dominant α-secretase pathway. This would directly and indirectly lead to excess neuroproliferation through multiple pathways, which would contribute to autism.

It is impossible to reduce ASDs to a direct, short-term etiology or collection of simple factors. Instead, etiology of multiple pathways and gene products probably underlie the condition. This is not to say that none of these could be critical. One such possible fundamental target would be APP at the nexus of neuroproliferation and neural pruning and its interaction with networks such as FMRP/mGluR, ERK/MAPK, and PI3K/mTOR (**Figure 2**). This presents a possible handle upon autism etiology, should sAPPα contribute to anabolic pathogenesis of the disorder. Of particular value is the evidence that APP dysfunction is more likely due to environmental/epigenetic interaction rather than strict genetic variation. Such dependency may permit environmental, e.g., dietary, remediation, as mentioned herein, and possible reversal at early stages of a disorder.

## Conflict of Interest Statement

The authors declare that the research was conducted in the absence of any commercial or financial relationships that could be construed as a potential conflict of interest.

## Abbreviations

AD: Alzheimer’s disease
ADAM: a disintegrin and metalloproteinase
AICD: APP intracellular domain
APP: amyloid-β precursor protein
ASD: autism spectrum of neurodevelopmental disorders
Aβ: amyloid-β peptide
FMR1: fragile X mental retardation 1 gene
FMRP: fragile X mental retardation protein
FXS: Fragile X syndrome
FXTAS: fragile X-associated tremor/ataxia syndrome
LEARn: latent early-life associated regulation
mGluR: metabotropic glutamate receptor; MRI, magnetic resonance imaging.
PI3K/mTOR: phosphoinositide 3 kinase/mammalian target of rapamycin
PKC: protein kinase C
PRKB1: protein kinase c-β gene
Ras/ERK: Ras small GTPase/extracellular signal-regulated kinase
sAPPα: soluble APP α
TACE: tumor necrosis factor-α converting enzyme
TIV: total intracranial volume
TNF-α: tumor necrosis factor α.
